# Social Cognition in Multiple Sclerosis: A 3-Year Follow-Up MRI and Behavioral Study

**DOI:** 10.3390/diagnostics11030484

**Published:** 2021-03-09

**Authors:** Stefano Ziccardi, Marco Pitteri, Helen M. Genova, Massimiliano Calabrese

**Affiliations:** 1 Neurology Section, Department of Neurosciences, Biomedicine and Movement Sciences, University of Verona, 37134 Verona, Italy; marco.pitteri@univr.it; 2 Kessler Foundation, 120 Eagle’Rock Ave, Suite 100, East Hanover, NJ 07936, USA; hgenova@kesslerfoundation.org; 3 Department of Physical Medicine and Rehabilitation, New Jersey Medical School, Rutgers University, Newark, NJ 07101, USA

**Keywords:** multiple sclerosis, social cognition, cognitive impairment, amygdala, psychological well-being

## Abstract

Social cognition (SC) has become a topic of widespread interest in the last decade. SC deficits were described in multiple sclerosis (MS) patients, in association with amygdala lesions, even in those without formal cognitive impairment. In this 3-year follow-up study, we aimed at longitudinally investigating the evolution of SC deficits and amygdala damage in a group of cognitive-normal MS patients, and the association between SC and psychological well-being. After 3 years (T3) from the baseline examination (T0), 26 relapsing-remitting MS patients (RRMS) were retested with a neuropsychological battery and SC tasks (theory of mind, facial emotion recognition, empathy). A SC composite score (SCcomp) was calculated for each patient. Emotional state, fatigue, and quality of life (QoL) were also evaluated. RRMS patients at T3 underwent a 3T-MRI as performed at T0, from which were calculated both volume and cortical lesion volume (CLV) of the amygdalae. Compared to T0, at T3 all RRMS patients were still cognitive-normal and remained stable in their global SC impaired performance. At T0, SCcomp correlated with amygdala CLV (*p* = 0.002) while, at T3, was more associated with amygdala volume (*p* = 0.035) rather than amygdala CLV (*p* = 0.043). SCcomp change T3-T0 correlated with global emotional state (*p* = 0.043), depression (*p* = 0.046), anxiety (*p* = 0.034), fatigue (*p* = 0.025), and QoL-social functioning (*p* = 0.033). We showed the longitudinal stability of SC deficits in cognitive-normal RRMS patients, mirroring the amygdala structural damage and the psychological well-being. These results highlight that SC exerts a key role in MS.

## 1. Introduction

Multiple sclerosis (MS) is a chronic immune-mediated inflammatory disease of the central nervous system, characterized by the formation of focal lesions of primary demyelination, both in the white matter (WM) and in the cortical and deep gray matter (GM), that progressively result in diffuse damage and neurodegeneration of the entire brain [1,2]. In the past twenty years, evidence identified GM alterations as a key component of the disease [3]: in particular, cortical lesions have been described as one of the pathological factors that could lead to the accumulation of cortical atrophy [4,5].

GM damage is significantly related to cognitive impairment (CI), one of the most frequent clinical symptoms in patients with MS: cortical lesions and atrophy commonly result in alterations regarding cognitive functioning [6,7] and CI also plays a prognostic role in predicting MS patients characterized by a higher risk of cortical atrophy progression [8]. The traditional cognitive assessment consists of neuropsychological batteries including tests that investigate the most frequent cognitive deficits in MS: learning and memory, information processing speed, attention, and executive functions [9].

In addition to the traditional neuropsychological assessment conducted with MS patients, both clinical and research interest is emerging around the concept of social cognition [10,11]. The term social cognition (SC) encompasses key mental operations underlying social interactions and processes required to establish and sustain interpersonal relationships [12]. SC is a multidimensional construct that involves different processes, including theory of mind, facial emotion recognition, and empathy [13]. Theory of mind (ToM) is defined as the ability to infer the intentions, dispositions, and beliefs of others [14], facial emotion recognition is the process of identifying human emotions from facial expressions [15], while empathy refers to the capacity to feel and understand what another person is experiencing [16]. Interest in the concept of SC has grown during the last twenty years, to such an extent that in 2013 SC was also included in the last revision of the Diagnostic and Statistical Manual of mental disorders (DSM-V) as one of the six key neurocognitive domains, together with learning and memory, complex attention, executive function, perceptual-motor function, and language. The earliest studies on SC were conducted on autistic patients [17] as SC difficulties are one of the main characteristics of this population. However, several recent studies reported SC impairment in neuropsychiatric disorders, such as schizophrenia [14,18,19], bipolar disorders [20,21,22], and major depressive disorders [23,24,25], as well as in evolutive conditions such as attention-deficit/hyperactivity disorder (ADHD) [26], in traumatic brain injuries cases [27], and in most of the neurodegenerative syndromes, such as Alzheimer’s disease [28,29], frontotemporal dementia [30,31], amyotrophic lateral sclerosis [32], and Parkinson’s disease [33,34] (see [35,36,37,38] for reviews conducted on different neurological populations).

Among patients suffering from neurological syndromes, studies on people with MS have been conducted in the last ten years to investigate SC functioning and its neural bases. Previous studies suggested the presence of SC impairment in MS patients in both relapsing and progressive forms of the disease [39,40], which can lead to the disruption of MS patients’ social environment and to a possible consequent reduction of their quality of life [41,42,43]. However, other studies did not report a significant difference between MS patients and a matched group of healthy controls in terms of SC performance [44,45,46]. The inhomogeneity of the results described in these studies may be addressed in the light of the cognitive reserve hypothesis, intended as higher levels of intellectual enrichment [47] that could mitigate the impact of brain damage on clinical outcomes, including cognitive and social cognitive functioning [10,13]. Moreover, the prevalence of deficits in SC performance could be mediated by possible confounders described as common symptoms in MS patients, such as fatigue, mood disorders, alexithymia, sleep problems, and CI [13,48].

In fact, one of the major discussion arguments in the MS literature refers to establishing whether SC deficits occur in association with cognitive alterations or not: some studies reported a significant association between CI and ToM [12,49,50,51,52,53], as well as facial emotion recognition [54,55,56] and empathy [50,57]. By contrast, other studies showed no correlation between CI and SC deficits in MS patients [41,42,58,59,60,61]. In order to contribute to this debate, a previous study by our group [62] overcame the correlation between CI and SC by directly investigating SC performance in a group of relapsing-remitting MS (RRMS) patients without evidence of formal CI. The results of our study showed that, despite being classified as “cognitively normal”, RRMS patients showed a significantly lower performance, compared to a matched group of healthy controls, in a task implying ToM, in a facial emotion recognition task (especially in negative emotions of fear and anger), and in a questionnaire investigating empathy [62]. Therefore, we concluded that RRMS patients were characterized by alterations in several SC domains, even in the absence of formal CI.

Moreover, we highlighted the specific role of left and right amygdala in explaining the results: the cortical lesion volume (CLV) in the bilateral amygdala was the only significant predictor of SC task performances among other global MRI parameters (cortical thickness, cortical lesions, WM lesion load) [62], supporting a core role of this deep brain structure in modulating social functioning in MS. However, it is not clear which amygdala parameter is the main predictor of SC performance in MS, since a previous study identified, instead, amygdala atrophy as the main predictor of the performance in a ToM test: a lower volume of the amygdala resulted in a poorer social performance [63].

To the best of our knowledge, to date all the studies regarding SC in MS patients were conducted using a cross-sectional approach; consequently, no study investigated the SC performance in MS patients with a longitudinal perspective. Studies conducted using a longitudinal design could evaluate the evolution of the variables of interest, in order to better clarify the cause-effect relationship between SC performance and structural amygdala damage, with observations performed at two different times, but in the same sample of patients. With the present study, we aimed to examine, after three years of follow-up, RRMS patients from our baseline study [62] in order to assess the evolution of SC deficits over time. Concurrently, we also aimed to investigate the evolution of amygdala lesion burden and atrophy after three years in the same sample of MS patients, and their association with SC performance. Furthermore, with respect to our baseline research [62], in this study we also introduced the evaluation of some everyday aspects regarding psychological well-being, such as emotional status, fatigue, and quality of life, in order to interpret SC functioning in a more ecological perspective.

## 2. Materials and Methods

Twenty-six RRMS patients (20 females, mean ± SD age = 39.5 ± 7.9 y, mean ± SD education = 12.8 ± 2.9 y, mean ± SD disease duration = 11.5 ± 5.0 y) were included in the present study as a sub-sample of a previous social cognition study [62] (5 patients from the baseline study were not retested since they no longer patients in our clinical center), in order to sustain a follow-up examination after an average period of 3.0 ± 0.2 years from the baseline. Compared to the baseline study, at the time of the follow-up examination all patients remained stable with the RRMS diagnosis (100%), while 4 patients (15%) were switched from a first-line to a second-line treatment. At the time of the present study, 1 patient received no specific treatment for MS, 14 were treated with dimethylfumarate, 5 with ocrelizumab, 4 with fingolimod, and 2 with natalizumab.

All participants were recruited and tested at the MS Center of the Verona University Hospital (Verona, Italy). The study was approved by the local Ethics Committee (protocol code 66,418, date of approval 25 November 2019) and informed consent was collected from all participants.

### 2.1. Cognitive and Psychological Assessment

All RRMS patients were tested with the same battery of neuropsychological tests used in our previous study [62], which included the Brief Repeatable Battery of neuropsychological tests (BRB; [64]) and the Stroop test (ST; [65]).

Scores below the cut-off (5th percentile) of the Italian normative data of each test were classified as failed. According to the most used method to consider CI in MS [66], RRMS patients were classified based on their performance in all the neuropsychological tests administered as “cognitively normal” (CN, up to 2 failed subtests) or “cognitively impaired” (CI, at least 3 failed subtests).

Furthermore, the emotional state (depression, anxiety, and stress) was evaluated by means of the Depression Anxiety and Stress Scale-21 (DASS-21; [67]), while fatigue was measured by using the Fatigue Severity Scale (FSS; [68]), and the quality of life was evaluated by means of the Multiple Sclerosis Quality of Life-29 (MSQoL-29; [69]).

### 2.2. Social Cognition Evaluation

We investigated the SC domain with the same protocol used and described in our previous study [62].

ToM was assessed by means of the Italian version of the Reading the Mind in the Eyes test (RME; [70]). The RME is composed of 36 black-and-white images of human faces (17 females and 19 males) representing the eyes of different individuals. Each image was shown on a screen for 5 s, then, on each corner of the image, 4 emotional adjectives were presented. Participants were asked to choose which of the 4 adjectives best described the emotion transmitted by each pair of eyes. As a control task, participants were also asked to indicate whether the eyes belonged to a female or a male face. The adjectives in each image shared the same valence, for example, “terrified”, “upset”, “arrogant”, and “annoyed”. One point was assigned for each correct response, while no points were assigned for incorrect responses.

Facial emotion recognition was assessed using the Task of Facial Emotion Recognition—Kessler Foundation (TOFER-KF, see [48] for details). The TOFER-KF is composed of 36 black-and-white photos of different actors displaying one of six basic emotions: happiness, anger, fear, sadness, surprise, and disgust. The stimuli were taken from the Karolinska Directed Emotional Faces (KDEF) database [71,72], which has been well validated. Each emotion was presented randomly on a screen six times (6 emotions × 6 times each = 36 stimuli). Participants were asked to choose which emotion each face was expressing. As a control task, participants were also asked to indicate whether each image was showing a female or a male face. One point was assigned for each correct response, while no points were assigned for incorrect responses.

The Empathy Quotient (EQ; [73]) is a self-reported questionnaire that participants were asked to fill in in order to measure their level of empathy. The EQ is composed of 60 items (40 about empathy and 20 about other domains), each with a 4-point Likert-scale as possible answers. For the 40 empathy questions, 21 were empathetic statements (0 points assigned to the answers “strongly disagree” and “slightly disagree”, 1 point assigned to the answer “slightly agree”, 2 points assigned to the answer “strongly agree”), while 19 were non-empathetic statements (0 points assigned to the answers “strongly agree” and “slightly agree”, 1 point assigned to the answer “slightly disagree”, 2 points assigned to the answer “strongly disagree”). No points were assigned to the 20 non-empathic questions, that served as control. The maximum total score is 80: the higher the total score, the higher the level of empathy.

A SC composite score (SCcomp) was calculated for each patient through a weighted average of the scores in the three SC tests, in order to have a global index of the SC domain, as previously described in other studies, both with MS patients [53] and in other clinical conditions [74,75]. Since for both RME and TOFER-KF the maximum score is 36, EQ scores were proportionally transformed from a maximum of 80 to a maximum of 36, and then all the scores of the three SC tests were averaged. This was applied both to baseline and follow-up data, in order to obtain a composite score change (ΔSCcomp) by subtracting the follow-up SCcomp to the baseline SCcomp.

### 2.3. MRI Acquisition

Each RRMS patient underwent a 3.0 T Phillips Achieva MRI (Phillips Medical Systems, Best, The Netherlands) with the same procedure as the baseline study [62]. The following sets of images were acquired:

- 3D fluid attenuated inversion recovery (FLAIR) repetition time (TR)/echo time (TE) = 5500/292 ms, inversion time (TI) = 1650 ms, voxel dimension of 1 × 1 × 1 mm^3^, matrix = 256 × 256;
- 3D double inversion recovery (DIR) TR/TE = 5500/292 ms, TI1/TI2 = 525/2530 ms voxel dimension of 1 × 1 × 1 mm^3^;
- 3D T1 weighted fast field echo (FFE) TR/TE = 8.4/3.7 ms, voxel dimension of 1 × 1 × 1 mm^3^, matrix = 256 × 256;
- 2D T1w spin echo Gadolinium (SE GD): TR/TE = 550/10 ms, 50 contiguous axial slices with a thickness = 3.0 mm, matrix = 256 × 256.

### 2.4. MRI Imaging Analysis

Each MRI has been evaluated by a neurologist with significant experience in MS, blinded to participants’ behavioral performance.

Cortical lesions (CLs) were evaluated on DIR images and corroborated on T1 weighted images, according to recent recommendations for CLs’ assessment in MS patients [76]. Cortical lesion volume (CLV) in the bilateral amygdala was calculated for each RRMS patient using a semiautomatic thresholding technique: each cortical lesion was previously manually identified and segmented, then the fuzzy C-mean algorithm [77] included in software developed at the National Institutes of Health (NIH), Medical Images Processing, Analysis and Visualization (MIPAV) (accessed on 26 February 2020 from http://mipav.cit.nih.gov), was applied to calculate the cortical lesion volume. The same procedure was used on FLAIR images to calculate WM lesions volume (WMLV).

Regarding morphometry, global cortical thickness and volume of the bilateral amygdala were calculated for each RRMS patient on the volumetric T1 weighted data set using the FreeSurfer image analysis suite [78], available online (accessed on 26 February 2020 from http://surfer.nmr.mgh.harvard.edu).

### 2.5. Statistical Analyses

Statistical analyses were performed using SPSS statistic software (SPSS Inc., Chicago, IL, USA, version 24), while figures and graphs were designed using GraphPad Prism (GraphPad, La Jolla, CA, USA, version 8).

To evaluate the cognitive status of MS patients, cognitive impairment classifications were compared between baseline and follow-up for each participant.

Paired t-tests were applied to compare social cognition performance between baseline and follow-up. Furthermore, depending on distribution normality, either Pearson or Spearman correlation analyses were carried out between SC and amygdala measures (lesions and volume), both at baseline and at follow-up, and also between change in SC performance and everyday measures (emotional state, fatigue, and quality of life). Moreover, stepwise regression models were performed using baseline and follow-up MRI data to predict follow-up SC performance.

Results were expressed as mean ± SD. A *p*-value less than 0.05 was considered significant.

## 3. Results

### 3.1. Cognitive Impairment

At the time of the baseline study, all the RRMS patients were classified as cognitively normal [62]; after an average follow-up of 3 years, considering the neuropsychological assessment performed, all RRMS patients (100%) showed no evidence of clinically relevant cognitive decline, then all of the participants were also classified as having no formal CI (cognitively normal, CN) at follow-up. Detailed results regarding neuropsychological performance of MS patients are reported in Table S1.

### 3.2. Social Cognition

Considering the SC performance, SCcomp and ΔSCcomp served as the dependent variables.

**Figure 1:**
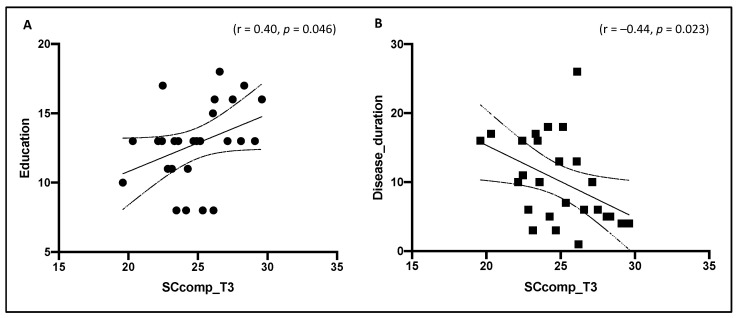
Association between SCcomp with education (**A**) and disease duration (**B**) at follow-up (T3). SCcomp = social cognition composite score.

At follow-up, we found a significant association for SCcomp with education (r = 0.40, *p* = 0.046) (Figure 1A) and disease duration (r = −0.44, *p* = 0.023) (Figure 1B): a higher performance in the SC tests is reflected by higher years of education and lower years of disease duration. However, no association was found between SCcomp and age (*p* = 0.28), and no difference in SCcomp was found between females and males (*p* = 0.97).

On the contrary, ΔSCcomp was not associated with demographical variables (education: *p* = 0.91, disease duration: *p* = 0.28, age: *p* = 0.43).

**Figure 2:**
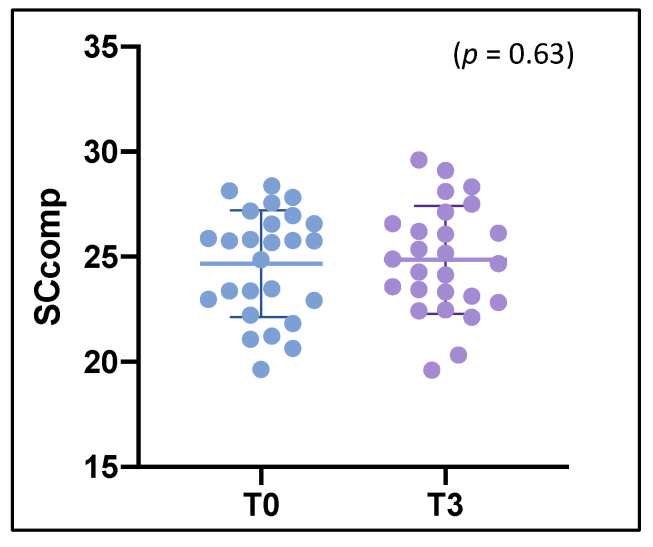
Comparison and evolution of SCcomp between baseline (T0) and follow-up (T3). SCcomp = social cognition composite score.

Regarding SCcomp longitudinal evolution, we did not find a significant difference between follow-up SCcomp (24.9 ± 2.6) and baseline SCcomp (24.7 ± 2.5) (*p* = 0.63) (Figure 2). Detailed results regarding SC performance of MS patients are reported in Table S1.

### 3.3. Social Cognition and MRI

As performed in our previous study [62], we evaluated the potential association between MRI parameters (WMLV, CLs number, global CTh, amygdala CLV) and the three SC tasks; results from the stepwise multiple regression analysis showed that amygdala CLV was the only significant imaging predictor for RME (R^2^ = 324, t(25) = −2.471, *p* = 0.023) and for TOFER-KF (R^2^ = 341, t(25) = −2.760, *p* = 0.012), while CLs number was the only significant predictor for EQ (R^2^ = 359, t(25) = −3.031, *p* = 0.007). Amygdala CLV also correlated with RME (r = −0.56, *p* = 0.004) and with TOFER-KF (r = −0.53, *p* = 0.006), in particular with negative emotions of fear (r = −0.44, *p* = 0.029) and sadness (r = −0.45, *p* = 0.023), while no correlation was found between amygdala CLV and EQ (*p* = 0.99). These results are in line with those described in our previous study [62], corroborating our baseline findings. Detailed results regarding MRI examinations are reported in Table S1.

**Figure 3:**
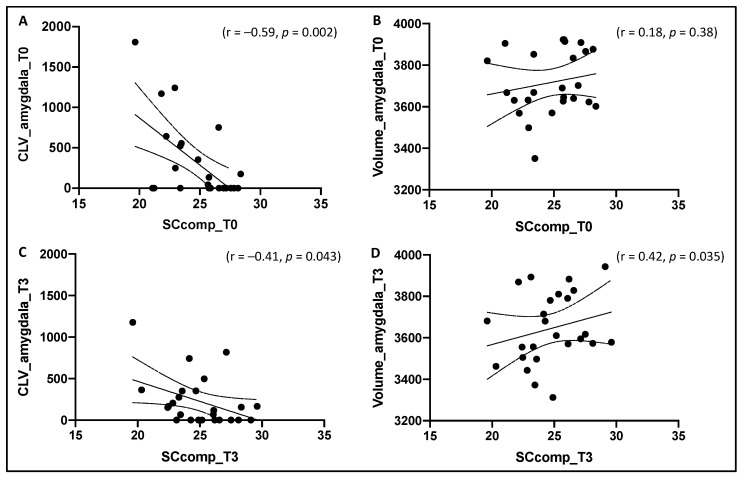
Association between SCcomp and, respectively, amygdala cortical lesion volume (CLV) (**A**) and amygdala volume (**B**) at baseline (T0), and between SCcomp and, respectively, amygdala cortical lesion volume (CLV) (**C**) and amygdala volume (**D**) at follow-up (T3). SCcomp = social cognition composite score.

Considering the SC global performance in association with amygdala examination, at baseline, SCcomp was significantly associated with the amygdala CLV (r = −0.59, *p* = 0.002) (Figure 3A); a higher amount of CLV in the bilateral amygdala reflected a lower performance in the SC tests. However, no association was found between SCcomp and the volume of the amygdala (*p* = 0.38) (Figure 3B). At follow-up, instead, SCcomp was significantly associated with both the amygdala CLV (r = −0.41, *p* = 0.043) (Figure 3C) and the volume of the amygdala (r = 0.42, *p* = 0.035) (Figure 3D); a lower SC performance was characterized by a higher amount of CLV in the amygdala and a lower volume of the amygdala itself.

Furthermore, stepwise regression models were performed using baseline and follow-up amygdala data and their association with follow-up SC performance. The model performed using baseline MRI data showed that CLV in the amygdala was the best predictor of SCcomp at follow-up (F = 4.3, *p* = 0.049). On the contrary, the model performed using follow-up MRI data showed that volume of the amygdala was the variable with the higher association with SCcomp at follow-up (F = 5.0, *p* = 0.035).

### 3.4. Social Cognition and Psychological Well-Being

**Figure 4:**
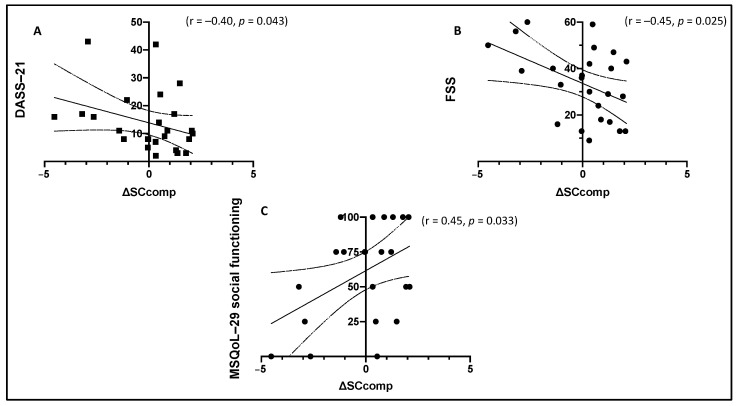
Significant association between ΔSCcomp and, respectively, DASS-21 global score (**A**), FSS global score (**B**), and MSQoL-29 social functioning (**C**). ΔSCcomp = change in social cognition composite score; DASS-21 = Depression Anxiety and Stress Scale-21; FSS = Fatigue Severity Scale; MSQoL-29 = Multiple Sclerosis Quality of Life-29.

Considering SC performance in association with psychological well-being, ΔSCcomp served as the dependent variable. We found a significant association between ΔSCcomp and DASS-21 outcomes, both for the global score (r = −0.40, *p* = 0.043) (Figure 4A) and for the single subscales of depression (r = −0.39, *p* = 0.046) and anxiety (r = −0.42, *p* = 0.034), suggesting that RRMS characterized by the greater worsening in SC performance were those more depressed and anxious at follow-up. Instead, no association was found with the DASS-21 stress subscale (*p* = 0.30). A significant association was also found between ΔSCcomp and FSS score (r = −0.45, *p* = 0.025) (Figure 4B); a greater worsening in SC performance was reflected by a higher physical fatigue at follow-up. Furthermore, we found a significant correlation between ΔSCcomp and the MSQoL-29 social functioning subscale (r = 0.45, *p* = 0.033) (Figure 4C), suggesting that RRMS characterized by the greater worsening in SC performance were those with a lower QoL regarding social functioning at follow-up, while we found no association between ΔSCcomp and both the MSQoL-29 global score and the other MSQoL-29 single subscales (all *p* > 0.05).

## 4. Discussion

With the present preliminary study, we aimed at investigating the longitudinal evolution of SC deficits, in parallel with the progression of lesion burden and atrophy accumulation in the bilateral amygdala, in a group of RRMS patients without evidence of formal cognitive impairment. We also aimed at evaluating the potential role of the social cognition domain on psychological outcomes of everyday life, such as emotional status, fatigue, and quality of life.

In the present study, we retested a group of RRMS patients, with a second time point evaluation, in order to corroborate the results obtained in our previous study with longitudinal data [62]. After an average period of three years since the previous examination, we observed a longitudinal consistency of both classification of CI and SC deficits; all RRMS patients were classified as being cognitively normal both at baseline and at follow-up (no evidence of clinically relevant cognitive decline), while SC performance after about three years showed the same level of impairment measured at baseline, with no additional worsening compared to the previous examination. A longitudinal stability of SC abilities was already reported in other neurological pathologies, such as schizophrenia [79], but to the best of our knowledge no data were provided for MS patients so far. These results could be due to the low sensitivity of CI classification criteria in MS patients; the use of a dichotomic criteria (“normal” vs. “impaired”) based only on traditional neuropsychological tests [80] might lead to underestimating cognitive alterations [81] and, therefore, social cognitive functioning. Traditional neuropsychological assessment seems to not be sufficient to fully estimate the cognitive status of MS patients, therefore it is necessary to also investigate the SC domain with specific tests.

Furthermore, it is known that MS is a neurological disease characterized by a gradual increase of neurodegeneration processes; however, the progression in the RR course typically requires several years and is revealed slower than the accelerated disease evolution in patients with progressive MS [82]. According to this, three years seems to be a relatively short period of time for our group of RRMS patients in order to show a further worsening in social cognition abilities, in addition to the one observed in the baseline study, so we can speculate that in our patients both compensatory mechanisms and cerebral plasticity are still not overpowered by pathological burden due to MS [83]. In fact, in this study we found a significant association between SC functioning and years of disease duration, suggesting that MS patients with a longer disease course showed higher SC deficits; however, since we also found an association between SC functioning and years of education, a possible regulatory effect of cognitive reserve could prevent a more rapid accumulation of severe SC impairment progression.

Moreover, in the present study we further explore the brain structural correlates of social cognition performance, deepening the investigation regarding the association between social tests and the amygdala that we already started in our previous study [62]. In particular, results of the present study reflected the association between amygdala CLV and SC performance found in our previous study [62], supporting the key role played by the amygdala in modulating SC performance, as previously demonstrated in other studies with MS patients [62,63,84]. However, we found that amygdala damage affected global SC functioning in different ways, on the basis of the time point considered. At baseline, in fact, SC global performance significantly correlated only with the amygdala CLV, while no association was found with the amygdala volume; nevertheless, at follow-up, SC global performances were associated with both amygdala lesion burden and atrophy, with the latter most associated with SC performance with respect to the former. It seems that cortical lesions in the amygdala could be the first mechanism that leads to early SC impairment [62] and is also the best predictor of SC global performance 3 years-later; however, after 3 years of follow-up, given that atrophy has been described as a probable pathological consequence of cortical lesions [4,5], amygdala atrophy had time to progress and therefore to be found associated with SC performance, even with the strongest correlation compared to the amygdala lesion burden.

The results of the present study also showed the significant association between SC performance and psychological aspects, realizing the SC domain into a fundamental process in order to conduct a peaceful daily life. Longitudinal change in the SC domain between baseline and follow-up resulted in being significantly associated with DASS-21, FSS, and MSQoL-29; in particular, RRMS patients with a longitudinal decrement in SC were also those patients characterized at follow-up by a higher level of depression and anxiety, a higher level of fatigue, and a lower level of quality of life regarding social functioning. Recent cross-sectional MS studies reported a significant association between SC and measures of depression, anxiety, fatigue [48], and quality of life [43]. Results of this follow-up study offered a real-life and ecological interpretation of the results (that often lacks in most of the studies already published), corroborating the idea that changes in SC are directly associated with emotional state, fatigue, and quality of life, and therefore could have an impact on these psychological aspects. Moreover, the specific association found between the SC domain and the social functioning sphere of the quality of life contributes to strengthening the validity of the results, creating a direct connection between the social abilities measured through clinical assessment and the actual social satisfaction of the patients in everyday life.

We are aware that this is a preliminary study that presents some limitations. Firstly, it was not possible to conduct an SC examination on the group of healthy controls included in the baseline study, which would have helped to obtain data from a control group to be compared with behavioral changes in RRMS patients. Secondly, the sample size is limited; further research should involve a higher number of MS patients to corroborate results from the present study and include patients with different characteristics (also progressive MS) such as disease duration, age, cognitive reserve, and disease-modifying therapies, that might affect the SC domain. Future studies should also include a more comprehensive MRI examination in order to investigate the contribution of other specific regions besides amygdala. Lastly, future research could benefit from using and validating more ecological and dynamic measures to assess the SC domain in individuals with MS.

## 5. Conclusions

The results of the present study showed that cognitively normal RRMS patients are characterized by a longitudinal stability in social cognition deficits after a period of three years, and that social cognition performance, at baseline and at follow-up, is associated differently with amygdala atrophy and cortical lesion burden. Longitudinal changes in the SC domain were found to be associated with psychological outcomes of daily living, such as depression, anxiety, fatigue, and social functioning quality of life. These results support the idea that the SC domain may exert a central role in MS patients that should be emphasized.

## Data Availability

The data presented in this study are available on request from the corresponding author.

## Supplementary Materials

The following are available online at https://www.mdpi.com/2075-4418/11/3/484/s1, Table S1: Clinical and brain MRI characteristics of the study participants.
