# Supplementary material for: Social Cognition in Multiple Sclerosis: A 3-Year Follow-Up MRI and Behavioral Study
Source: Diagnostics (Basel). 2021 Mar 9;11(3):484. doi: 10.3390/diagnostics11030484 (PMC8001246; doi:10.3390/diagnostics11030484)
Supplement: Supplementary file 1 [file diagnostics-11-00484-s001.pdf]

	Patient factor	Subcomponent	Raw scores T0	Raw scores T3
**Cognitive assessment**	BRB-NT	SRT-LTS	51.4±11.7	59.2±10.4
		SRT-CLTR	42.0±14.3	52.6±13.1
		SRT-D	9.8±1.7	11.0±1.7
		SPART-I	23.9±4.2	24.9±3.6
		SPART-D	8.3±1.7	8.6±1.2
		SDMT	56.0±7.8	58.1±9.3
		PASAT-3	46.1±8.9	51.1±7.4
		PASAT-2	35.4±9.7	40.8±10.0
		WLG	26.7±4.5	28.7±5.5
	ST	ST-EIT	12.5±5.0	12.6±4.7
		ST-EIE	0.4±1.0	0.3±0.6
**Psychological assessment**	Emotional state	DASS-21 D	/	3.7±4.1
		DASS-21 A		2.8±3.3
		DASS-21 S		6.7±4.9
		DASS-21 TOT		13.4±11.0
	Fatigue	FSS-TOT	/	33.6±15.4
	Quality of life	MSQoL-29 PF	/	67.8±34.5
		MSQoL-29 BP		68.5±22.5
		MSQoL-29 EW		61.0±18.9
		MSQoL-29 E		48.2±16.7
		MSQoL-29 CF		71.2±18.1
		MSQoL-29 HD		77.7±20.6
		MSQoL-29 SeF		71.4±32.9
		MSQoL-29 SoF		60.9±34.4
		MSQoL-29 HP		43.5±25.5
		MSQoL-29 CH		52.2±23.7
		MSQoL-29 OQoL		68.3±15.3
		MSQoL-29 PHC		61.2±18.3
		MSQoL-29 MHC		63.5±17.7
**Social cognition (SC) evaluation**	Facial emotion recognition	TOFER-KF	28.0±3.4	28.8±2.7
	Theory of mind	RME	25.4±3.1	26.6±3.0
	Empathy	EQ	46.0±10.0	42.3±10.2
	Global SC	SCcomp	24.7±2.5	24.9±2.6
**Neurological evaluation**	Physical disability	EDSS *(median and IQR)*	1.0 (0-3.0)	2.0 (1.0-3.0)
**MRI acquisition**	Global MRI measures	WMLV *(cm^3)*	5.1±2.0	5.5±2.3
		CLs number	7.4±5.5	8.1±5.6
		Global CTh *(mm)*	2.4±0.3	2.3±0.2
	Amygdala	Amygdala volume *(mm^3)*	3717.7±154.5	3644.6±171.0
		Amygdala CLV *(mm^3)*	306.1±485.0	226.8±302.0

Table S1. Clinical and brain MRI characteristics of the study participants. BRB-NT = Brief Repeatable Battery of Neuropsychological Tests, SRT-LTS = Selective Reminding Test – Long Term Storage, SRT-CLTR = Selective Reminding Test – Consistent Long Term Retrieval, SRT-D = Selective Reminding Test – Delayed, SPART-I = Spatial Recall Task – Immediate, SPART-D = Spatial Recall Task – Delayed, SDMT = Symbol Digit Modalities Test, PASAT-3 = Paced Auditory Serial Addition Task – 3 seconds, PASAT-2 = Paced Auditory Serial Addition Task – 2 seconds, WLG = Word List Generation, ST = Stroop Test, ST-EIT = Stroop Test – Effect Interference Time, ST-EIE = Stroop Test – Effect Interference Error, DASS-21 D = Depression Anxiety and Stress Scale – 21 Depression, DASS-21 A = Depression Anxiety and Stress Scale – 21 Anxiety, DASS-21 S = Depression Anxiety and Stress Scale – 21 Stress, FSS = Fatigue Severity Scale, MSQoL-29 PF = Multiple Sclerosis Quality of Life – 29 Physical Function, MSQoL-29 BP = Multiple Sclerosis Quality of Life – 29 Bodily Pain, MSQoL-29 EW = Multiple Sclerosis Quality of Life – 29 Emotional Well-being, MSQoL-29 E = Multiple Sclerosis Quality of Life – 29 Energy, MSQoL-29 CF = Multiple Sclerosis Quality of Life – 29 Cognitive Function, MSQoL-29 HD = Multiple Sclerosis Quality of Life – 29 Health Distress, MSQoL-29 SeF = Multiple Sclerosis Quality of Life – 29 Sexual Function, MSQoL-29 SoF = Multiple Sclerosis Quality of Life – 29 Social Function, MSQoL-29 HP = Multiple Sclerosis Quality of Life – 29 Health Perception, MSQoL-29 CH = Multiple Sclerosis Quality of Life – 29 Change in Health, MSQoL-29 OQoL = Multiple Sclerosis Quality of Life – 29 Overall Quality of Life, MSQoL-29 PHC = Multiple Sclerosis Quality of Life – 29 Physical Health Composite, MSQoL-29 MHC = Multiple Sclerosis Quality of Life – 29 Mental Health Composite, TOFER-KF = Task of Facial Emotion Recognition – Kessler Foundation, RME = Reading the Mind in the Eyes test, EQ-60

= Empathy Quotient, SCcomp = Social Cogniton composite score, EDSS = Expanded Disability Status Scale, WMLV = white matter lesion volume, CL = cortical lesions, CTh = cortical thickness, CLV = cortical lesion volume.
